# Zebrafish Insights into Nanomaterial Toxicity: A Focused Exploration on Metallic, Metal Oxide, Semiconductor, and Mixed-Metal Nanoparticles

**DOI:** 10.3390/ijms25031926

**Published:** 2024-02-05

**Authors:** Chinmaya Mutalik, Nivedita, Chandrasekaran Sneka, Dyah Ika Krisnawati, Sibidou Yougbaré, Chuan-Chih Hsu, Tsung-Rong Kuo

**Affiliations:** 1 Graduate Institute of Nanomedicine and Medical Engineering, College of Biomedical Engineering, Taipei Medical University, Taipei 11031, Taiwan; cm121193@tmu.edu.tw; 2 International Ph.D. Program in Biomedical Engineering, College of Biomedical Engineering, Taipei Medical University, Taipei 11031, Taiwan; d845111002@tmu.edu.tw (N.); d845111006@tmu.edu.tw (C.S.); 3 Department of Nursing, Faculty of Nursing and Midwifery, Universitas Nahdlatul Ulama Surabaya, Surabaya 60237, East Java, Indonesia; dyahkrisna77@gmail.com; 4 Institut de Recherche en Sciences de La Santé/Direction Régionale du Centre Ouest (IRSS/DRCO), Nanoro BP 218, 11, Burkina Faso; ysibidou@gmail.com; 5 Division of Cardiovascular Surgery, Department of Surgery, School of Medicine, College of Medicine, Taipei Medical University, Taipei 11031, Taiwan; 6 Division of Cardiovascular Surgery, Department of Surgery, Taipei Medical University Hospital, Taipei 11031, Taiwan; 7 Stanford Byers Center for Biodesign, Stanford University, Stanford, CA 94305, USA

**Keywords:** nanomaterial, metal, semiconductor, zebrafish, toxicology, ROS

## Abstract

Nanomaterials are widely used in various fields, and ongoing research is focused on developing safe and sustainable nanomaterials. Using zebrafish as a model organism for studying the potentially toxic effects of nanomaterials highlights the importance of developing safe and sustainable nanomaterials. Studies conducted on nanomaterials and their toxicity and potential risks to human and environmental health are vital in biomedical sciences. In the present review, we discuss the potential toxicity of nanomaterials (inorganic and organic) and exposure risks based on size, shape, and concentration. The review further explores various types of nanomaterials and their impacts on zebrafish at different levels, indicating that exposure to nanomaterials can lead to developmental defects, changes in gene expressions, and various toxicities. The review also covers the importance of considering natural organic matter and chorion membranes in standardized nanotoxicity testing. While some nanomaterials are biologically compatible, metal and semiconductor nanomaterials that enter the water environment can increase toxicity to aquatic creatures and can potentially accumulate in the human body. Further investigations are necessary to assess the safety of nanomaterials and their impacts on the environment and human health.

## 1. Introduction

Nanomaterials have been the subject of intense research for several decades, and they are now used in a wide range of fields, including electronics, medicine, materials science, and energy [1,2,3,4,5]. Due to their unique physical and chemical properties, nanomaterials have been widely investigated for use in biomedicine and biotechnology, including drug delivery, antibiotics, antibacterial agents, bioimaging, biofuels, and tissue engineering [6,7,8]. One of the reasons for the growing interest in nanomaterials is their unique properties, which can significantly differ from those of larger-scale materials. For example, dendrimers have flexible dimensions and sizes; hence, they can be used in drug-delivery systems [9]. Nanodiamonds are functionally convenient and biocompatible; thus, they can be used in drug design, gene transfer, etc. [10]. However, the use of nanomaterials also raises concerns about their potential impacts on human health and the environment, particularly given their small size and large surface area. Ongoing research is focusing on developing safe and sustainable nanomaterials and exploring their potential benefits in various fields [11].

Zebrafish (*Danio rerio*) is a widely used model organism in biomedical and biotechnological research, including for fluorescence and bioimaging studies [12]. Their small size, transparency during the early stages of embryonic development, and rapid development make them ideal for studying the structure and function of living organisms at cellular and molecular levels [13]. Features of zebrafish illnesses, development, and molecular mechanisms are clinically relevant and largely conserved among vertebrates, making them an important preclinical model for human diseases. They can be utilized for high-throughput and high-content phenotypic drug screening, drug repurposing, and even the creation of new drug classes because they respond to small compounds and therapeutic therapies at physiologically appropriate dose ranges. Zebrafish are sophisticated, complete animals that can be investigated at a single-cell precision level, making it possible to track drug activity throughout tissues and over long periods. Drugs and drug leads with interorgan modes of action that would not otherwise be found using specific screening methods have been found using zebrafish. Future options for exploiting zebrafish’s potential in medical discoveries are highlighted. Zebrafish have significant promise for drug discovery [14]. Nanosized drug delivery systems, like Doxil^®^, improve drug properties and combat resistance. These systems, effective in various cancers, particularly sarcomas, combined with zebrafish models, offer a promising approach for better cancer prognosis and toxicology assessment [15,16,17,18,19,20].

Various applications of the use of nanomaterials in zebrafish research have shown promise [21,22,23]. For instance, gold nanoparticles (AuNPs) have been used to deliver therapeutic agents, such as drugs or small interfering (si)RNA, to specific cells or tissues in zebrafish embryos [24]. Silver (Ag) NPs have also been utilized as an antibacterial agent in zebrafish larvae [25]. Carbon nanotubes have been used to study the toxicity of nonmaterial in zebrafish embryos [26]. Additionally, the use of zebrafish in nanomaterials research has also helped to better understand the potentially toxic effects of NPs on living organisms. For instance, researchers found that some NPs can cause developmental abnormalities in zebrafish embryos or lead to changes in gene expressions [22].

Zebrafish can be genetically modified to express fluorescent proteins that allow researchers to visualize specific cells, tissues, or processes in vivo. For example, fluorescent proteins can be targeted to specific cell types, such as neurons or blood vessels, to study their development and function [27]. In addition to genetic modifications, zebrafish can also be labeled with fluorescent dyes or NPs to visualize specific structures or processes. For example, quantum dots and other NPs can be conjugated to biomolecules, such as antibodies or peptides, that bind to specific targets in the body, allowing for targeted imaging and toxicity evaluation in zebrafish [28]. Bioimaging techniques such as confocal and two-photon microscopy can capture high-resolution images of zebrafish tissues and organs in real time [29]. These techniques allow researchers to study dynamic biological processes, such as cell migration, morphogenesis, and disease progression, in living organisms.

In the present review, the zebrafish model, in combination with metallic, metal–semiconductor, and mixed-metal nanomaterials, is shown to have great potential for advancing biomedical and biotechnological research. Zebrafish can be widely used in a range of research fields, including developmental biology, genetics, neuroscience, drug discovery, and fluorescence and bioimaging studies, due to their transparency, genetic tractability, and physiological similarity to humans [30].

## 2. Metal Nanoparticles

Inorganic, organic, and carbon-based materials are the three main categories into which nanomaterials are classified [31]. Metal NPs are considered inorganic nanomaterials and are highly sought-after for their widespread use in biomedical science. These NPs can be easily synthesized and modified with functional groups, allowing them to be conjugated with antibodies, ligands, and drugs of interest, leading to a vast range of potential applications. Metal NPs such as Ag, Au, palladium (Pd), titanium (Ti), zinc (Zn), and copper (Cu) are commonly used as carriers for drug delivery, transporting therapeutic agents like antibodies, nucleic acids, chemotherapeutic drugs, peptides, etc. In addition, these NPs possess advanced optical properties and have many applications across various research fields [32]. Although metal NPs are widely utilized, it is crucial to examine their potential risks and toxicity to both humans and aquatic ecosystems. Several studies have shown that zebrafish are a promising animal model for assessing NP toxicity due to their many advantageous characteristics, including a short lifespan and high reproductive capacity [33]. Various studies exhibited diverse reactions, and disparities among them can be attributed to various factors such as variations in the tested materials’ characteristics (such as particle size and surface coating), discrepancies in NP concentrations, the use of different cell models, variations in testing protocols, discrepancies in genotoxicity endpoints, and differences in exposure durations [34].

**Figure 1:**
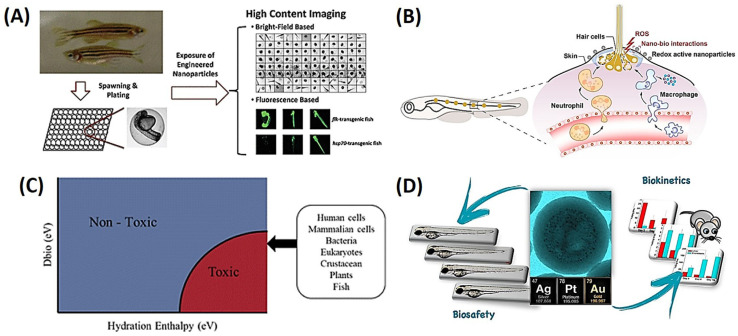
(**A**) High-content bright-field imaging reveals robust and dose-dependent interference with hatching in embryos, with the notable exception of cobalt oxide (Co_3_O_4_), which exhibited relative inertness. Reproduced with permission from ref. [36], American Chemical Society 2011. (**B**) Redox-active nanoparticles (NPs) caused skin damage and triggered the infiltration of immune cells in zebrafish embryos. Reproduced with permission from ref. [37], American Chemical Society 2020. (**C**) An in silico assessment of the toxicity of metal and metal oxide NPs across various cell types and organisms. Reproduced with permission from ref. [38], Royal Society of Chemistry 2023. (**D**) Gold, silver, and platinum ultrasmall-in-nano architectures. Reproduced with permission from ref. [39], American Chemical Society 2019.

The article utilizes zebrafish models to assess the safety of engineered nanomaterials (ENMs). To compare the harmful effects of several transition metal oxide NPs (copper oxide (CuO), zinc oxide (ZnO), nickel oxide (NiO), and cobalt oxide (Co_3_O_4_)) on zebrafish embryos and larvae, the researchers used high-content bright-field and fluorescence-based imaging [35]. They discovered that some NPs interfered with hatching in a dose-dependent manner. This was probably caused by the loss of Cu and Ni ions, which compromised zebrafish hatching enzyme 1 (ZHE1). However, there were no apparent morphological anomalies. Additionally, certain NPs caused transgenic zebrafish larvae to express more heat shock protein 70 (HSP70), which can be used for hazard ranking and dose–response analyses. In general, HSP70 expression and high-content imaging of embryo development can be a useful technique for determining the safety of ENMs in zebrafish, as shown in Figure 1A [36]. An article covers the potential for harm to biological systems from redox-active metal oxide NPs and includes studies utilizing zebrafish as a model organism to examine the biological injury potential and processes of two metal oxide libraries, Co_3_O_4_ and palladium oxide (PdO)-Co_3_O_4_. The research discovered that Cu dopants increased Co_3_O_4′_s ability to oxidize, increased the mortality of dechorionated embryos, and increased the severity of skin damage in exposed larvae. Additionally, more PdO led to larger heterojunction densities, which raised the oxidizing capability. The NPs caused epidermal damage and triggered immune cell infiltration, which led to an inflammatory reaction. The skin injury reduced the tolerance level against other environmental pollutants, even if exposure to the NPs alone was not fatal. However, the skin damage in larvae was temporary and reversible because they were able to recover in uncontaminated media in just 24 h, as shown in Figure 1B [37]. Gakis et al. discussed increasing concerns over the impacts of engineered NPs on humans, animals, and ecosystems and the development of in silico methods such as structure–activity relationship (SAR) models to rapidly screen for toxicity. The authors redirected their attention to extracting valuable mechanistic insights from models by employing a substantial dataset comprising 935 toxicity measurements of 45 metal and metal oxide nanoparticles (NPs). They constructed classification nanostructure–activity relationship (nano-SAR) models, which effectively categorized NPs according to their toxicity to different cells and organisms, thereby identifying potential mechanisms of toxicity. The proposed approach sought to steer researchers towards the development of nanoparticles designed for safety and foster more informed risk assessments of nanoparticles, as illustrated in Figure 1C [38].

Another study discussed the need for regulations for engineered nanomaterials to protect people and prevent possible lawsuits. The authors conducted a meta-analysis of three nanomaterials to compare their toxicity in nano and dissolved forms. The results showed that, in most cases, the nanoform was less toxic than the dissolved metal form based on the total metal concentration. Therefore, decreasing the current regulatory metal concentration thresholds for ecotoxicity by a factor of two may potentially offer adequate protection for organisms against the nanoform of these metals as well [40]. Due to their persistence and the paucity of information on their biokinetics, the efficient excretion of nanostructured noble metals presents a problem for their use in clinical practice. In zebrafish and mouse models, the biosafety and biokinetics of gold, silver, and platinum ultrasmall-in-nano structures were quantitatively connected [39]. These metals, despite being of similar sizes, utilize various excretion paths due to their inherent metallic compositions. For noble-metal NPs to be used clinically for treating neoplasms, infectious illnesses, and neurological disorders, it is crucial to comprehend their in vivo fate, as shown in Figure 1D [39].

### 2.1. Gold Nanoparticles

**Figure 2:**
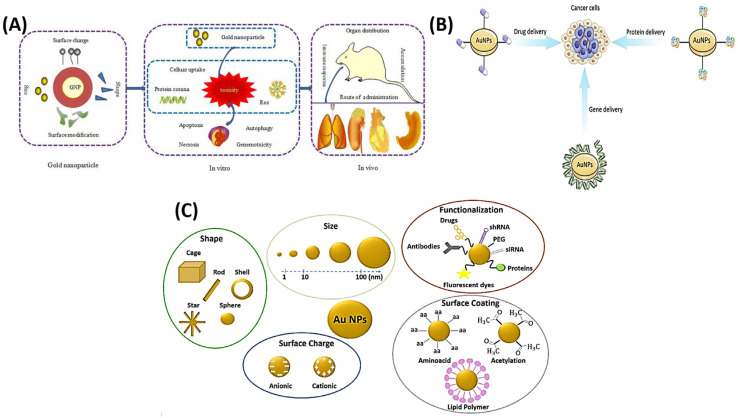
(**A**) Schematic representation of in vitro and in vivo toxicity of gold nanoparticles (AuNPs). Reproduced with permission from ref. [46], Elsevier 2017. (**B**) Application of delivery carriers for AuNPs. Reproduced with permission from ref. [49], Frontiers Media S.A. 2020. (**C**) Different factors influencing AuNPs’ toxicity include their shape, size, functionalization, surface charge, and surface coatings. Reproduced with permission from ref. [30], MDPI 2021.

AuNPs are widely used in biomedical fields due to their advantageous properties such as tunable sizes, easy synthesis, and strong optical properties [41,42,43,44]. They have been utilized for various applications, including immunochemical studies, DNA fingerprinting, antibiotic detection, cancer diagnosis, and bacterial identification [45]. In Figure 2A, AuNPs also exhibit in vivo toxicity and can damage organs such as the spleen, liver, and kidneys [46]. Despite this, their unique electronic properties and physical properties make them useful for medical diagnostic and therapeutic applications [46,47,48]. Hu et al. discussed the potential medical applications of AuNPs due to their unique functional properties and easy synthesis [49]. Those authors summarized the various methods of synthesis, modification, and characterization of AuNPs, as well as their potential applications in drug and gene delivery, photothermal therapy, photodynamic therapy, radiation therapy, and diagnosis. The article serves as a comprehensive reference for future studies on medical applications of AuNPs, as shown in Figure 2B [49]. In other studies, exposure of zebrafish embryos to small spherical AuNPs (with a size range of 12~35 nm) had no significant impact on their development [30]. The mortality rate was low, there were no delays in hatching, and no changes were observed in the heartbeat rate, even at the tested concentrations. Embryos were carefully evaluated for any abnormalities after being anesthetized with 0.1% phenoxyethanol and observed under a microscope. At 24 h post-fertilization (hpf), treated embryos and larvae exhibited normal development of the eyes, otoliths, brain, and tail. Furthermore, there were no perturbations observed in pigmentation or organs at 48 or 72 hpf. Conversely, larger AuNPs (86 nm) resulted in higher mortality rates and a greater percentage of morphological abnormalities than smaller ones (12~35 nm). This finding demonstrated a direct and proportional correlation between the size of AuNPs and their toxicity. It is worth noting that compared to other similarly sized plasmonic NPs, AuNPs were found to be less toxic to zebrafish during development, as shown in Figure 2C [30]. Patibandla et al. examined the potential toxic effects of NPs, specifically the effects of gold nanospheres and different types of gold nanorods on zebrafish embryos [50]. Their study found that exposure to gold nanorods and polystyrene sulfate-coated gold nanorods resulted in significant increases in mortality and decreases in hatching and heart rates, as well as changes in expressions of oxidative stress genes. However, these effects were reduced when the NPs were coated with polystyrene sulfate and polyallamine hydrochloride. Gold nanospheres were found to be the least toxic.

AuNPs have been increasingly explored as drug carriers for efficient skin drug delivery due to their unique properties and versatility. The skin barrier can be overcome by NP-based drug carriers to improve the delivery of even hydrophilic or macromolecular drugs. Factors that contribute to the penetration behavior of AuNPs, such as size, surface chemistry, and shape, were summarized in a review [51]. The design of AuNPs for dermal or transdermal drug delivery was implicated in drug loading, release, and penetration patterns. Physical methods like microneedles and ionophoresis were presented as effective means to enhance the delivery efficacy of AuNPs. The paper suggested that AuNPs hold significant promise as drug carriers, which can be used to design multifunctional systems for drug delivery through the skin. As a result, this review emphasized the significance of gold nanoparticles (AuNPs) in delivering drugs to the skin and offered direction for future research exploring AuNPs as a versatile and efficient approach to drug delivery.

### 2.2. Silver Nanoparticles

AgNPs have different applications in medicine, agriculture, and other fields due to their unique properties at the nanoregime in comparison with that of bulk gold [52,53,54]. AgNPs have tunable physical and chemical properties that make them suitable for improving the activity of drugs, developing sensors for detecting biomarkers and pollutants, and generating electrocardiographs. An article highlighted the different approaches used to prepare AgNPs, including physicochemical and biological methods, each with its own pros and cons [55]. However, excessive use of AgNPs can be cytotoxic and may have adverse effects on the environment. This article concluded that research into AgNPs has always been driven by the need to develop a technology with potential benefits and minimal risk to environmental and human health. The finding suggests that the exploration of AgNPs has consistently been motivated by the pursuit of technology advancement that offers promising advantages while maintaining a keen focus on mitigating potential risks to both the environment and human health.

AgNPs have potential biomedical applications due to their unique properties, including antimicrobial activity, drug-delivery capabilities, and tissue regeneration properties. There has been significant interest in using AgNPs to enhance personalized healthcare practices. However, there is also concern about the potential toxicity of these NPs. The latest research examined biomedical uses of AgNPs and their potential toxic effects, as well as their intrinsic anti-inflammatory, antibacterial, antiviral, and antifungal activities [56].

**Figure 3:**
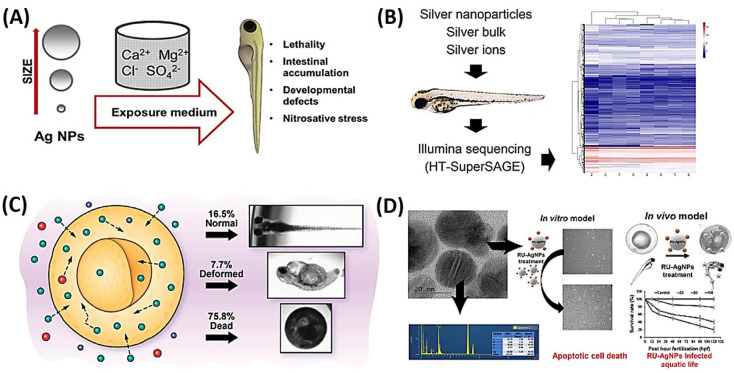
(**A**) Intestinal toxicity of three different sizes (10, 40, and 100 nm) of silver nanoparticles (AgNPs) in embryonic zebrafish, and descriptions of relationships of properties and behaviors of AgNPs in the exposure medium with the induction of lethal and sublethal effects. Reproduced with permission from ref. [57], Elsevier 2019. (**B**) Assessing the consequences of silver exposure in nano, bulk, and ionic states on zebrafish embryos (*Danio rerio*) utilizing next-generation sequencing techniques. Reproduced with permission from ref. [58], American Chemical Society 2013. (**C**) Toxic effects on embryonic development dependent on AgNP dosage and size. Reproduced with permission from ref. [59], American Chemical Society 2012. (**D**) In vitro and in vivo analysis of *Rumex acetosa* (RU)−AgNPs. Reproduced with permission from ref. [60], Elsevier 2019.

Liu et al. discussed the intestinal toxicity of different sizes of AgNPs (10, 40, and 100 nm) in embryonic zebrafish and the factors that contribute to their toxic response [57]. That study found that the size and composition of the exposure medium contributed to differential NP agglomeration, the release of Ag ions, and subsequent effects during exposure. Lethality was primarily observed for embryos exposed to medium-sized AgNPs (40 nm), while exposure to smaller and larger NPs and Ag ions only caused sublethal effects, as shown in Figure 3A [57]. Another study found that AgNPs are toxic to adult zebrafish, with gills being the primary route of exposure, internalization, and toxicity [30]. Chronic exposure to AgNPs led to their accumulation in various organs and the treatment-induced concentration-dependent perturbations in gene expressions associated with different biological processes. AgNP internalization affected ion regulatory functions and disturbed the flux of all ions, leading to disturbances in osmotic balance. Treated zebrafish exhibited high levels of stress hormones and low levels of electrolytes in their plasma. Additionally, adult zebrafish treated with chemically produced NPs and biologically synthesized AgNPs exhibited signs of respiratory toxicity, with a significant increase in the respiratory rate. These findings suggest that AgNPs have the potential to affect the health of adult zebrafish and may impact the wider aquatic ecosystem due to their bioaccumulation and biomagnification properties. A study looked at how zebrafish embryos responded to exposure to silver in ionic, bulk, and nanoforms [58]. With the aid of next-generation sequencing, the researchers discovered substantial changes in gene expressions with each treatment. Strong overlaps in gene pathways impacted by the three therapies suggested comparable toxicity mechanisms. After 24 h of exposure, oxidative phosphorylation patterns showed downregulation in this system, which recovered after 48 h. The results were significant in assessing the risk that AgNPs may pose to exposed vertebrate creatures because they confirmed the idea that the toxicity caused by AgNPs is primarily linked to the accessibility of silver ions in exposed zebrafish eggs, as shown in Figure 3B [58].

A study investigated the transport and toxicity of AgNPs in early-developing zebrafish embryos [59]. Nanomaterials are attractive for many applications because of their distinctive physicochemical characteristics, yet they can also have severe side effects. Pure and stable AgNPs were created in this study, and it was discovered that they passively diffuse into embryos and remain there throughout their development. The size and dose-dependence of the NPs’ toxicity were observed, with larger AgNPs being more hazardous than smaller ones at the same molar concentration. Compared to normal zebrafish, deformed zebrafish had a greater number of larger NPs implanted in their tissues. This discovery raised the prospect of modifying AgNPs’ biocompatibility and toxicity, as shown in Figure 3C [59]. The use of AgNPs as antimicrobial agents in commercial applications is widespread, but there are growing concerns about their potential environmental and health effects. To investigate their impacts and defense mechanisms at the molecular level, zebrafish embryos were subjected to continuous exposure to two sizes of AgNPs of 4 and 10 nm, from 4 to 96 hpf [61]. The results showed that exposure to 4 nm AgNPs had a significant effect on zebrafish embryos by 72 hpf. These findings highlight the importance of particle size as a potential factor in the toxicity development of AgNPs in fish embryos.

The development of toxicity of AgNPs toward zebrafish embryos was studied, and it was found that exposure to AgNPs caused developmental defects and altered gene expressions related to DNA replication, cellular senescence, and oxidative phosphorylation pathways [62]. Additionally, AgNP exposure resulted in the accumulation of reactive oxygen species (ROS) and malondialdehyde, inhibition of enzyme activities, and downregulation of genes related to mitochondrial function and apoptosis. These findings suggested that AgNP exposure can cause oxidative stress, mitochondrial dysfunction, and toxicity development in zebrafish embryos. 

*Rumex acetosa* (RU)-AgNPs and their toxicity toward human umbilical vein endothelial cells (HUVECs) and zebrafish embryos were investigated through a green method. RU-AgNPs induced the formation of ROS in HUVECs, leading to apoptosis in a dose-dependent manner. In zebrafish, RU-AgNPs caused mortality, malformation, an imbalanced heart rate, and severe morphological changes, accompanied by increases in apoptotic biomarkers and ROS. The study suggested that RU-AgNPs can cause toxicity via ROS-induced apoptotic pathways, indicating the need to assess toxicity levels before using them in biomedical applications, as shown in Figure 3D [60].

### 2.3. Copper Nanoparticles

Copper is one of the most common elements and plays an important role in the normal functioning of organisms. CuNPs have unique properties that are promising for various applications in electronics, biomedicine, and agriculture [63]. In addition to their antibacterial capabilities, CuNPs were found to possess antifungal, antiviral, and anticancer properties, making them superior to current antibiotics [64]. Potential ecological risks related to the usage of Cu-based NPs in commercial and industrial products are covered in an article [36]. The paper examines the aquatic toxicity of Cu and CuO NPs as well as NP dissolution and organismal uptake of these NPs. The toxicity of these NPs can vary based on their physicochemical properties. The study examines the fate and ecotoxicological effects of Cu and CuO NPs in a mixed aquatic environment using a multispecies microcosm. The findings implied that both NPs are ingested by living things and cause toxicity by particle-specific mechanisms. Overall, the study emphasizes how crucial it is to take into account the environmental impacts of copper-based NPs when using them in different products [65]. Another study used chorion-intact (CI) or dechorionated (DC) embryonic zebrafish to examine how the chorion affects the toxicity of copper-based NPs [66]. They discovered that the chorion prevented Cu toxicity and that when the chorion was removed, embryo sensitivity increased by at least an order of magnitude. CuNPs and CuO NPs differ in toxicity in part because of their capacity to produce ROS, which should be taken into account in risk evaluations.

**Figure 4:**
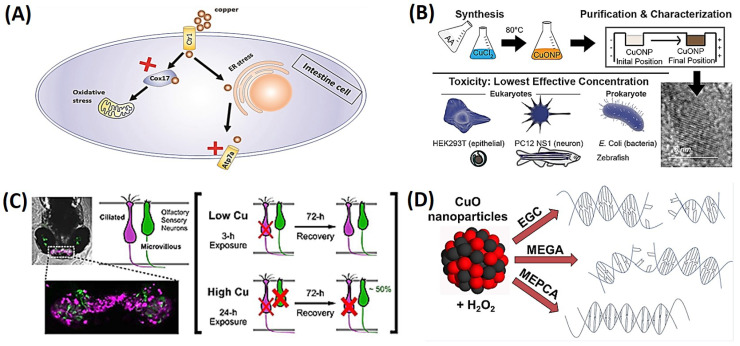
(**A**) Exposure to copper nanoparticles (CuNPs) and Cu^2+^ induced intestinal defects in zebrafish, as per the study. Inhibition of copper transportation to mitochondria or the trans−Golgi network (TGN) did not alleviate the reactive oxygen species (ROS) or endoplasmic reticular (ER) stress-induced defects. Reproduced with permission from ref. [67], Oxford Academic 2020. (**B**) Comparative study evaluating biocompatibility and cytotoxicity of sub−10 nm CuNPs across multiple biological systems, including *Escherichia coli*, yeast, HEK293T and PC12 cell lines, and zebrafish embryos. Reproduced with permission from ref. [69], American Chemical Society 2020. (**C**) Confocal imaging of double−transgenic zebrafish larvae reveals olfactory sensory neuron (OSN) differentiation using differential labeling of ciliated and microvillus OSNs. Reproduced with permission from ref. [70], Elsevier 2018. (**D**) Cupric oxide NPs and H_2_O_2_−mediated DNA damage and cytotoxicity effects. Reproduced with permission from ref. [71], Elsevier 2022.

Zhao et al. investigated the impact of CuNPs and the released Cu^2+^ on intestinal development in zebrafish embryos. The results revealed that exposure to elevated levels of CuNPs or Cu^2+^ led to damage in intestinal development in a dose-dependent manner. CuNP exposure resulted in a reduction in the expression of intestinal marker genes while increasing the expression of endoplasmic reticular (ER) stress marker genes in zebrafish intestines. Immunofluorescence analysis indicated that both CuNPs and Cu^2+^ induced the production of ER stress and oxidative stress in intestinal cells. The inhibition of ER stress or oxidative stress using 4-phenylbutyric acid or ROS scavengers restored the expression of intestinal marker genes. Interestingly, blocking the transport of copper to mitochondria or the trans-Golgi network did not alleviate copper-induced intestinal developmental defects. In summary, this study demonstrated that CuNPs and Cu^2+^ induce intestinal developmental defects by triggering ER and ROS stress, as depicted in Figure 4A [67,68].

The biocompatibility and cytotoxicity of sub-10 nm CuNPs to several biological systems, such as yeast, mammalian cell lines, prokaryotic cells, and zebrafish embryos, were investigated [69]. To eliminate the partial toxicity effect from leftover reactants, a purification method utilizing agarose gel electrophoresis was devised. The findings demonstrated that throughout the course of 18 h treatment, pure CuNPs suppressed bacterial growth and killed eukaryotic cells at respective concentrations of 170 and 122.5 ppm (*w*/*w*). The pigmentation of the retinal pigmented epithelium of zebrafish embryos at 85 ppm was likewise markedly decreased by the NPs. According to the study, both prokaryotic and eukaryotic systems can be damaged by tiny CuNPs; hence, care should be taken to prevent direct contact with human tissues, considering their potential use in clinical settings, as shown in Figure 4B [69].

The olfactory system of zebrafish larvae, which primarily rely on their sense of smell for survival, was examined in a study to determine how copper exposure affected it [70]. The death and regeneration of olfactory sensory neurons (OSNs) in response to copper exposure were examined by researchers using confocal imaging. They discovered that exposure to copper led to a variety of morphological alterations in both ciliated and microvillus OSNs, including cell death and fragmentation, axon loss, disorderly cell configurations, and a decrease in the intensity of the fluorescence signal. At low copper concentrations, ciliated OSNs were lost more severely than microvillus OSNs, although microvillus OSNs recovered more quickly than ciliated OSNs. The rise in bromodeoxyuridine labeling suggested that olfactory stem cells had taken the place of injured OSNs. Olfactory behavioral assessments, which showed early loss and restoration of olfactory function after copper exposure, similarly validated the imaging investigations. This study emphasized the vulnerability of fish olfactory systems to environmental toxins and the ability of microvillus OSNs to regenerate after damage, as shown in Figure 4C [70]. Another study looked at the capacity of 10 polyphenolic antioxidants to guard against DNA damage and cytotoxicity caused by CuO NPs and H_2_O_2_. Three of the polyphenols had no effect, two enhanced DNA damage, and five demonstrated preventative effects against DNA damage. The study also discovered that in the presence of CuO NPs or free copper ions, the majority of polyphenols exhibited comparable antioxidant/prooxidant activities. Methyl 3,4-dihydroxybenzoate inhibited the generation of ROS, while vanillic acid had no impact, and epigallocatechin elevated ROS levels, according to electron paramagnetic resonance spectroscopy. The study discovered that under oxidative stress, polyphenols might shield DNA and prevent cytotoxicity caused by CuO NPs, as shown in Figure 4D [71]. The effect of adding CuNPs to a universal adhesive system used for etch-and-rinse (ER) and self-etching (SE) techniques was evaluated [72]. In total, 216 restorations were placed in 36 subjects, and the restorations were evaluated at the baseline and at 6, 12, and 18 months. The addition of CuNPs did not increase the clinical performance of the adhesive in the ER mode, but it significantly improved the retention rate and decreased marginal discrepancies in the SE mode after 18 months. This study provided evidence for a slight improvement in the clinical performance of universal adhesive systems in noncarious cervical lesions with the addition of lower concentrations of CuNPs.

Rajeshkumar et al. conducted a study on the extracellular synthesis of copper sulfide (CuS) NPs using *Aeromonas hydrophila* and explored their various biological applications, including antibacterial, anti-inflammatory, and antioxidant activities. The researchers characterized the synthesized NPs using several analytical techniques such as UV-visible spectrophotometry, Fourier transform infrared (FTIR) spectroscopy, atomic force microscopy (AFM), and scanning electron microscopy (SEM). Additionally, the toxicological effects of CuS NPs were assessed using zebrafish as an animal model. The effects of excess copper (in the form of CuNPs and Cu ions) on retinal development in zebrafish embryos resulting from copper exposure led to retinal cell death, downregulation of retinal genes, and damage to the ER and mitochondria in retinal cells. The study also found that the upregulation of unfolded protein responses and ROS contributed to the copper-induced retinal defects. However, retinal developmental defects were significantly neutralized using ROS scavengers and ER stress inhibitors. Additionally, blocking copper transportation by deleting the *cox17* and *atp7a* genes alleviated retinal developmental defects in embryos under copper stress [73].

The ability of the olfactory mucosa to regenerate throughout life is well known, but exposure to neurotoxic contaminants like CuNPs and Cu ions can hinder this process. A study on rainbow trout olfactory mucosa found that CuNPs inhibited neuron regeneration and reduced expressions of proinflammatory proteins required for neuroregeneration. Gene expressions involved in axonal regrowth mainly increased after CuNP treatment, but a few cell adhesion molecules and neural polarity genes were downregulated. Cu^2+^ treatment initiated both neural repair strategies and led to recovery from olfactory dysfunction. Overall, CuNPs had a greater impact on olfactory repair mechanisms than Cu^2+^ [74].

### 2.4. Platinum Nanoparticles

**Figure 5:**
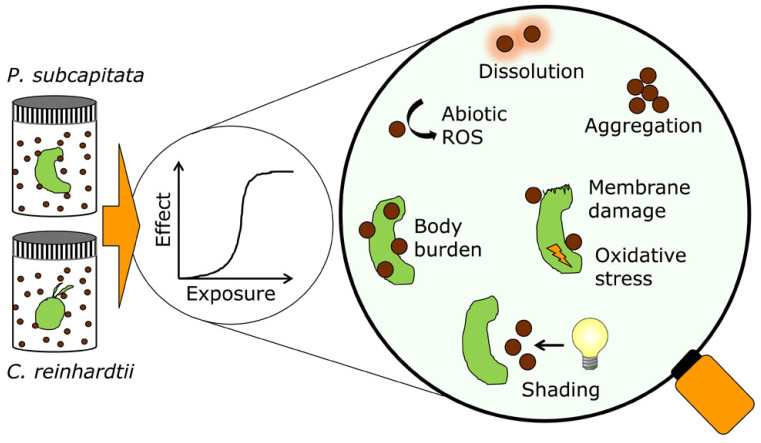
Characterization techniques and toxicity endpoints to investigate platinum nanoparticles (PtNP) toxicity toward the green microalgae *Pseudokirchneriella subcapitata* and *Chlamydomonas reinhardtii*. Reproduced with permission from ref. [76], American Chemical Society 2016.

Due to their distinctive catalytic capabilities and substantial surface area, PtNPs are used in a variety of biotechnological, nanomedical, and pharmaceutical applications. The synthesis, characterization, and biomedical use of PtNPs are all thoroughly discussed in this paper, along with their physical, chemical, biological, and toxicological consequences on human health. The article also explores the uses of PtNPs in medicine and diagnostics, as well as the usage of diverse templates for their biological synthesis [75]. PtNPs are often found in the form of a suspension or colloid of PtNPs in a fluid, typically water. A colloid refers to a stable dispersion of particles in a fluid medium, either liquid or gas. Under specific reaction conditions, spherical PtNPs can be synthesized with sizes ranging from 2 to 100 nm, resulting in a suspension with a maroon or black color. NPs come in various shapes such as spheres, rods, cubes, and tetrahedra. PtNPs have garnered significant attention for their potential applications in several areas, including catalysis, medicine, and the synthesis of unique materials with special properties. Using various methodologies and toxicity endpoints, the toxicity of PtNPs—frequently employed in automotive catalytic converters—was studied on two species of microalgae. *Pseudokirchneriella subcapitata* was found to be more susceptible to PtNPs than *Chlamydomonas reinhardtii* and to have a higher body load, but both algal species experienced growth rate suppression and oxidative stress as a result of PtNPs. The study emphasized how crucial it is to employ several strategies in order to comprehend the workings and byproducts of NP-ecotoxicity testing, as shown in Figure 5 [76].

### 2.5. Bimetallic Nanoparticles

#### 2.5.1. Gold–Silver Nanoparticles

**Figure 6:**
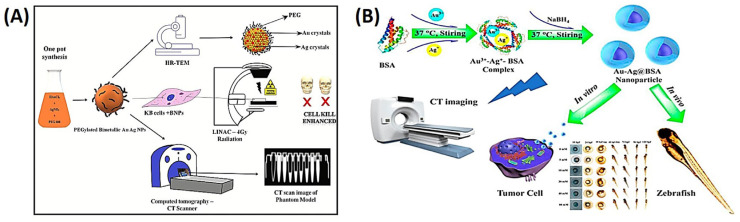
(**A**) Bimetallic intermixed nanoparticles demonstrated dual functionality as radiosensitizers and computed tomographic (CT) contrast agents, effectively targeting and treating oral cancers and other forms of cancer. Reproduced with permission from ref. [77], Dovepress 2021. (**B**) Schematic Illustration of the fabrication and application of gold–silver (Au-Ag)@bovine serum albumin (BSA) nanoparticles. Reproduced with permission from ref. [78], American Chemical Society 2019.

The use of PEG-coated Au-Ag alloy NPs as a possible radiosensitizer and computed tomographic (CT) contrast agent against oral malignancies in a recent study is discussed in a recent article [77]. Polyethylene glycol (PEG) 600 was used in NP synthesis as a reducing and stabilizing agent. Nuclear magnetic resonance (NMR), small-angle X-ray scattering (SAXS), Raman spectroscopy, and electron microscopy were used to characterize the NPs. The bimetallic NPs (BNPs) showed strong in vitro radiosensitization with an enhanced ratio of 1.5–1.7 and were shown to infiltrate the cytoplasm of KB cells. Apoptosis was visible through imaging of Hoechst-stained nuclei in a dose-dependent manner. The study also showed that BNPs were more effective at enhancing CT contrast than Omnipaque. Overall, the bimetallic intermixed NPs may be used to treat mouth cancer by acting as both a radiosensitizer and a CT contrast agent, as shown in Figure 6A [77]. Another research described the environmentally friendly synthesis of ultrasmall Au-Ag BNPs (2~4 nm), directed by bovine serum albumin (BSA), with well-dispersed ability and long-term durability. The study modified several molar ratios of Au-Ag to examine the effects of synthesis circumstances. Compared to individual AgNPs, the resultant NPs exhibited superior biocompatibility and decreased cytotoxicity. A reasonable design of the Au/Ag molar ratio (3:2) with better CT performance was highlighted in the study, which also examined the in vivo toxicity of NPs in early-stage zebrafish embryos. This design could be helpful in the development of CT contrast agents, catalysts, and drug delivery vehicles, as shown in Figure 6B [78].

#### 2.5.2. Ruthenium–Ferrocene

**Figure 7:**
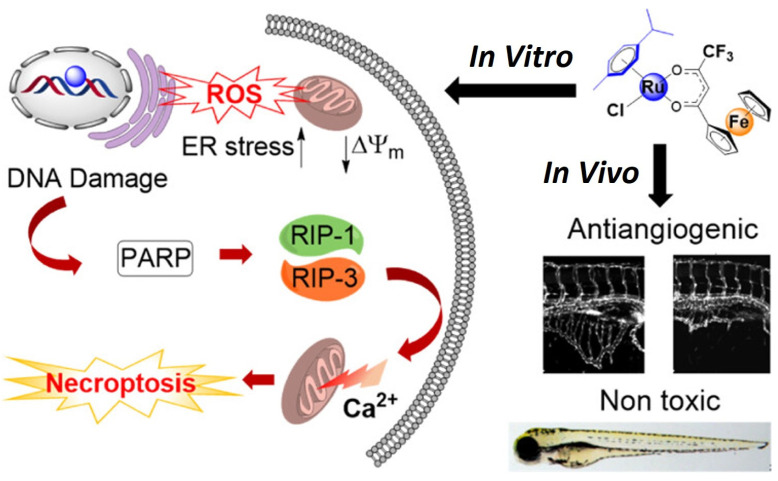
Intracellular toxicity pathway after nanomaterial interaction. Reproduced with permission from ref. [79], American Chemical Society 2022.

One recent study showed that resistance and adverse effects of platinum anticancer medications were addressed by combining the pharmacophores of ruthenium and ferrocene to create a bimetallic anticancer agent [55]. The agent’s cellular absorption, intracellular distribution, and effects on nucleophilic biomolecules were assessed, along with its in vivo antiangiogenic capabilities, Pt cross-resistance profile, and anticancer efficacy. According to research, the substance caused mitochondrial malfunction, ER stress, and necroptotic cell death mediated by poly(ADP-ribose) polymerase by producing ROS. In order to comprehend the effects of various groups on the anticancer potency of the Ru-Fc hybrid, the mechanism of action is shown in Figure 7 [79].

### 2.6. Metal–Semiconductor Nanoparticles

#### 2.6.1. Iron–Zinc Oxide Nanoparticles

**Figure 8:**
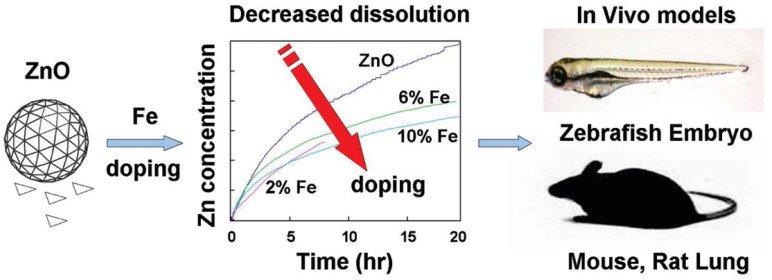
In vitro and in vivo effect of zinc oxide (ZnO) doped with iron (Fe). Reproduced with permission from ref. [80], American Chemical Society 2011.

A recent study demonstrated the usage of iron (Fe)-doped ZnO NPs to lessen the harmful effects of ZnO NPs in vivo [80]. In cellular screens, it was discovered that the doped particles had lower rates of dissolution and toxicity. The doped particles reduced harmful effects such as inflammatory cell infiltrates, cytokine levels, and lactate dehydrogenase (LDH) release in rodent and zebrafish models. According to the study, iron doping could be a secure design technique to avoid ZnO toxicity in both animals and the environment, as shown in Figure 8 [80].

#### 2.6.2. Cobalt and Cobalt Oxide Nanoparticles

Zebrafish embryos and human colon cell lines were used as models to examine the nanotoxicity of Co_3_O_4_ NPs [81]. The medicinal plant, *Calotropis gigantea,* was used in a green method to create the NPs. The research discovered that Co_3_O_4_ NP exposure enhanced oxidative stress and apoptosis in both cell lines and embryos, with commercial C-CoO NPs showing higher amounts of these effects than green-produced G-CoO NPs. Co_3_O_4_ NPs interacted with proteins implicated in oxidative stress and apoptosis, causing dysregulation of their structural and functional integrity, according to an in silico study. The work emphasized how crucial intrinsic atomic interactions between amino acids and Co_3_O_4_ NPs were in determining their cellular biocompatibility [81].

## 3. Semiconductor- and Carbon-Based Nanomaterials

NPs are compounds with a size of fewer than a hundred nanometers. Their small size allows them to have several unique properties that differ from one another, and these materials have been found in nature. There are various kinds of NPs. Unique ones can be found in common waterways, soils, or volcanic wastes produced by biological and geological cycles. NPs are increasingly being used in the medical, pharmaceutical, cosmetic, and food industries [4,5]. The increased use of NPs and their byproducts may be hazardous to living creatures. A survey of European consumers found that NPs were present in over 2300 commercial products. Titanium dioxide (TiO_2_), aluminum oxide (Al_2_O_3_), silicon oxide (SiO_2_), carbon nanotubes, ZnO, ferrous oxide, ceric oxide, Au, and CuS are common NPs [82,83,84,85,86,87]. The majority of nanoparticles (NPs) exhibit biocompatibility and chemical inertness, with examples such as gold and titanium dioxide [88]. These NPs serve as valuable research tools and find widespread use in various industries, including medicine, construction, agriculture, and the production of consumer goods such as electronics, packaging, cosmetic products, and textiles. Titanium dioxide nanoparticles (TiO_2_ NPs) are particularly recognized for their high catalytic activity, which is attributed to their small size, enabling a larger surface area per unit mass [89]. Nanoparticles (NPs) have the potential to enter aquatic ecosystems, and metal NPs, when introduced into the water environment, can exhibit toxicity towards aquatic organisms like fish, impacting the food chain. Human exposure to metal and metal oxide NPs may occur through the consumption of contaminated water or the ingestion of contaminated organisms such as vegetables, fish, and animals. This raises concerns about the deposition of these nanoparticles in the human body and potential risks to human health [90]. To address these concerns, there is a need for the development of new tools and methods capable of assessing both exposure levels and the toxicity of these nanomaterials.

The zebrafish animal model during both adult and embryonic stages has become popular in toxicology and biomedical research. The reason for zebrafish’s widespread popularity as an animal model is due to their unique properties: they are very small, they rapidly develop, have high reproducibility and transparency, and can be used for embryo-proven genetic and chemical screening. Zebrafish require inexpensive housing, making them cost-effective. It was observed that zebrafish are small-sized animals; hence, they can be handled with little difficulty. The rapid hatching of eggs and initiation of feeding by larvae at 120 h post-fertilization (hpf) are notable advantages of zebrafish embryos as a model. Their transparency facilitates easy identification of malformations, which are key indicators for toxicity assessments. Malformations may include embryonic and organ abnormalities, incomplete development of body parts like the head, eyes, or tail, anomalies such as a bent notochord, fin deformities, and lack of pigmentation—examples of developmental malformations. Even if nanoparticles (NPs) are unable to penetrate cells due to size constraints, they can still disrupt membrane activities, such as signal transduction and ion transport, by interacting with cell membranes. The physical and chemical characteristics of NPs, including positive electric charges, can be harmful, damaging membrane lipid bilayers. The surface coating of NPs significantly influences cell structure. Moreover, the impacts of NPs can be influenced by various contaminants, as they have the capacity to absorb hazardous elements that may be toxic to organisms. This is particularly relevant in aquatic organisms within the food chain, and harm to these creatures can disrupt the entire chain. Semiconducting NPs, with their unique optical and electronic properties, are attractive for diverse applications. Studies have demonstrated that exposure of zebrafish to semiconducting nanoparticles leads to an increase in reactive oxygen species (ROS) [91]. However, their increasing use in consumer products and medical applications has raised concerns about their potential toxicity. Studies have shown that semiconducting NPs can induce oxidative stress, inflammation, and cell death in vitro and in vivo.

**Figure 9:**
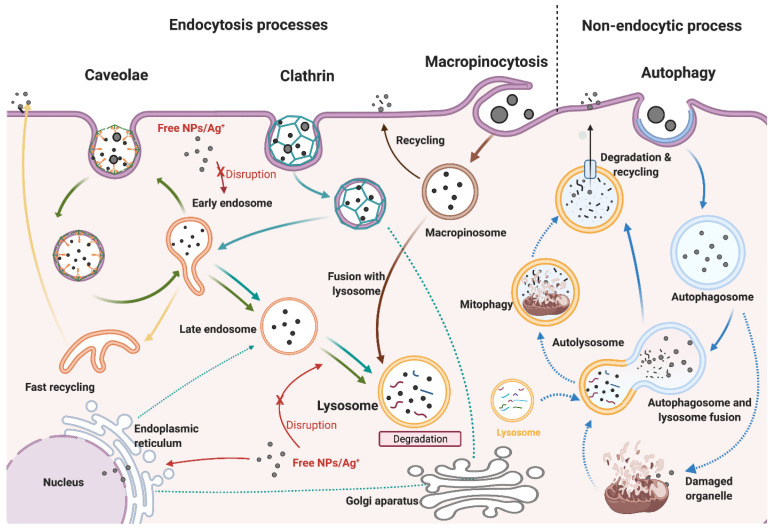
Mechanisms of cellular uptake by the chorion membrane of zebrafish. Reproduced with permission with ref. [92], MDPI 2021.

Cellular uptake is a fundamental biological process employed by cells, necessitating various transport mechanisms to facilitate the ingestion of macroparticles from their surroundings. This process is collectively referred to as receptor-mediated endocytosis, encompassing phagocytosis and pinocytosis. Phagocytosis entails cells engulfing particulate matter for subsequent digestion, while pinocytosis relies on vesicles to internalize fluids and other molecules, depending on the specific molecular interactions triggered. Both caveolin-dependent endocytosis and clathrin-dependent endocytosis lead to the formation of early endosomes, which are endocytic vesicles. Caveolin-dependent endocytosis, in particular, involves the internalization of external materials within flask-shaped vesicles through receptor binding on cell membranes and their corresponding external targets. Conversely, micropinocytosis involves the creation of large vesicles capable of internalizing nonspecific cargoes and substantial quantities of fluid. Consequently, the extent of cellular uptake can vary depending on the particle size of the nanoparticle, as illustrated in Figure 9 [92].

### 3.1. Titanium Dioxide Nanoparticles

**Figure 10:**
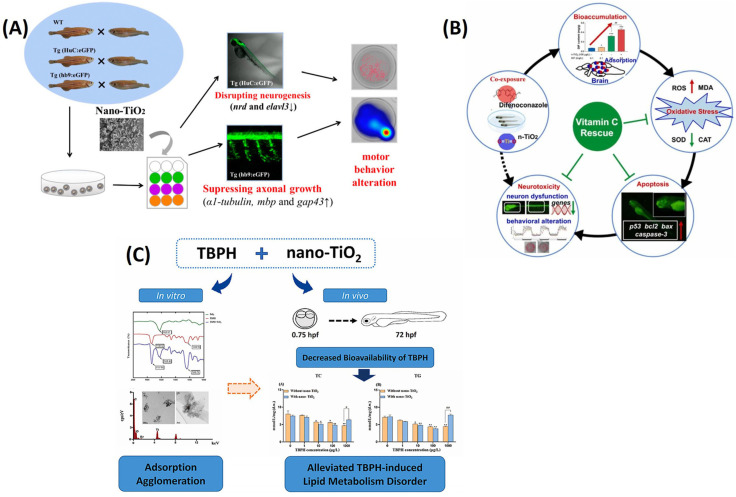
(**A**) Exposure to nano-TiO_2_ results in DNA damage and adverse neural outcomes in zebrafish larvae, inhibiting neurodevelopment and motor neuron axonal growth. Reproduced with permission from ref. [95], Elsevier 2021. (**B**) Exposure to high levels of nano-TiO_2_ inhibits neurodevelopment and motor neuron axonal growth in zebrafish larvae, leading to adverse neural outcomes and altered gene expression. Reproduced with permission from ref. [96], Elsevier 2023. (**C**) Co-exposure of TBPH and nano-TiO_2_ reduces toxicity in zebrafish embryos due to larger agglomerates. Reproduced with permission from ref. [97] Elsevier 2022.

The ecotoxicity of TiO_2_ NPs has become a major concern due to their widespread use in various fields. Although previous studies indicated that TiO_2_ NPs have neurotoxic effects, the underlying mechanism remains largely unknown [93,94]. In the mentioned study, zebrafish embryos underwent exposure to varying concentrations of TiO_2_ nanoparticles (NPs) and micro-TiO_2_ for a duration of up to 6 days post-fertilization. The outcomes of this investigation unveiled that exposure to TiO_2_ NPs at a concentration of 1.0 mg/L led to a significant reduction in both the body length and weight of zebrafish larvae. Remarkably, hatching rates and mortality remained unaffected by this exposure. Furthermore, behavioral assessments conducted in the study indicated that exposure to TiO_2_ NPs had a noticeable impact on the swimming behavior of the larvae. Specifically, it resulted in a significant reduction in swimming speed and a decrease in the time spent in clockwise rotations among the larvae [95]. Additional investigation demonstrated that exposure to TiO_2_ NPs had detrimental effects on the length of motor neuron axons in Tg (hb9-GFP) zebrafish and hampered the process of central nervous system (CNS) neurogenesis in Tg (HuC-GFP) zebrafish. A real-time polymerase chain reaction (PCR) analysis also showed significant alterations in genes associated with neurogenesis and axonal growth due to TiO_2_ NP exposure. This study provided compelling evidence that early-life exposure of zebrafish to TiO_2_ NPs led to adverse neural outcomes by impeding neurodevelopment and motor neuron axonal growth, as illustrated in Figure 10A. Additionally, DNA damage was observed using a COMET assay, a technique for measuring DNA damage in individual cells. It involves electrophoresis of cells in an agarose gel and visualizing the resulting comet-like image.

The widespread use of TiO_2_ NPs in various fields has raised concerns about their ecotoxicity, particularly neurotoxicity, although the underlying mechanism remains unclear. To shed light on this issue, zebrafish embryos were exposed to varying concentrations of TiO_2_ NPs and micro-TiO_2_ for up to 6 days after fertilization. The results showed that exposure to high levels of TiO_2_ NPs had significant adverse impacts on the body length and weight of zebrafish larvae, as well as their swimming speed and clockwise rotation times. However, there were no significant effects on hatching or mortality rates of embryos. Further analysis demonstrated that TiO_2_ NP exposure inhibited neurodevelopment and motor neuron axonal growth, leading to adverse effects on CNS neurogenesis in Tg (HuC-GFP) zebrafish and motor neuron axon length in Tg (hb9-GFP) zebrafish [98]. A real-time PCR analysis also revealed that TiO_2_ NP exposure significantly affected genes associated with neurogenesis (*nrd* and *elavl3*) and axonal growth (*α1-tubulin*, *mbp*, and *gap43*). In summary, the study highlighted the adverse neural outcomes associated with early-life exposure of zebrafish to TiO_2_ NPs, which was attributed to the inhibition of neurodevelopment and motor neuron axonal growth, as shown in Figure 10B [96].

It is believed that NPs such as TiO_2_ NPs may affect the way that coexisting organic or inorganic pollutants behave in aquatic environments, potentially increasing their toxicity. Recently, a new type of flame retardant called bis(2-ethylhexyl)-2,3,4,5-tetrabromophthalate (TBPH) was found in many environments, including those where TiO_2_ NPs were also present. However, it is not clear how TBPH and TiO_2_ NPs interact with each other in water or how they affect biological processes and toxicity at environmentally relevant levels. To investigate this, zebrafish embryos were exposed to TBPH alone or with TiO_2_ NPs, and their interactions were studied in terms of physicochemical properties and variations in bioavailability and toxicity with regard to lipid metabolism. In vitro tests showed that TBPH and TiO_2_NPs adsorbed and agglomerated together. The bioavailability of TBPH was reduced when it was exposed to TiO_2_ NPs, as indicated by decreased body contents of both substances. Additionally, lipid metabolism disorders caused by TBPH alone were mitigated when it was co-exposed with TiO_2_ NPs [98]. These results suggested that TBPH and TiO_2_ NPs form larger agglomerates when they are adsorbed together, leading to decreased bioavailability and reduced toxicity with regard to lipid metabolism in developing zebrafish embryos and larvae, as shown in Figure 10C [97].

### 3.2. Zinc Oxide Nanoparticles

**Figure 11:**
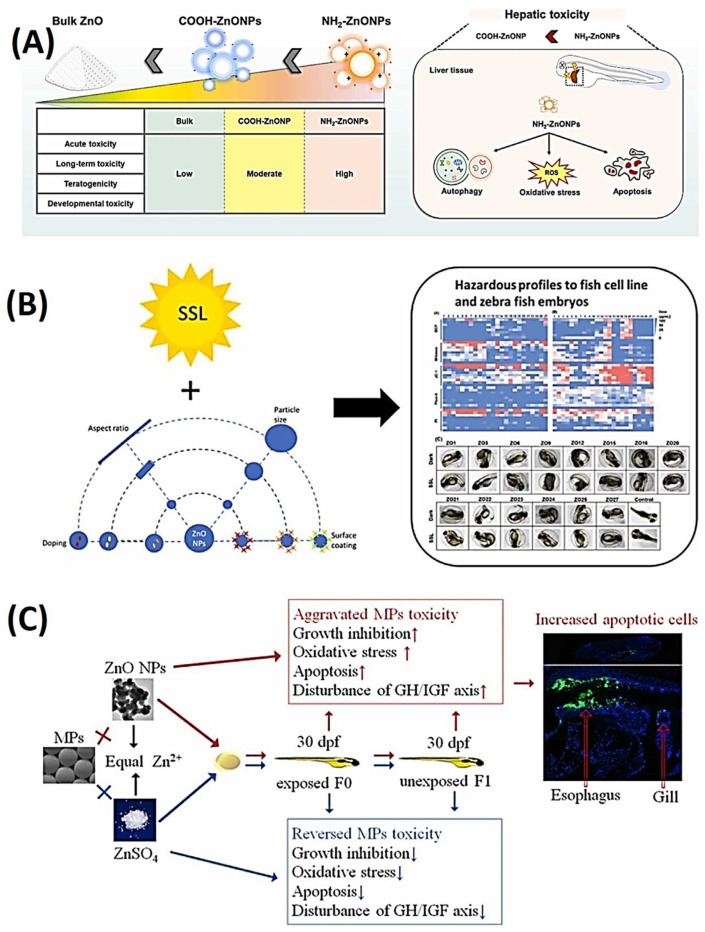
(**A**) NH_2_-coated zinc oxide nanoparticles (ZnO NPs) induce a reactive oxygen species (ROS) burden, activate autophagy-related genes, and ultimately lead to liver cell apoptosis and reduced sizes of developing zebrafish. Reproduced with permission from ref. [91], Elsevier 2022. (**B**) Light exposure amplifies ZnO NP toxicity, but surface coatings mitigate the effect, as revealed by a fish cell line and zebrafish embryos. Reproduced with permission from ref. [101], Elsevier 2022. (**C**) Microplastics (MPs) and ZnO NPs interact to increase toxicity in zebrafish larvae, with ZnO particles adhering to MP surfaces, leading to increased Zn transport into larvae. The effects were further exacerbated in F0 larvae by co-exposure to MPs and ZnO NPs and in F1 larvae exposed to MPs ^+^ ZnO. Reproduced with permission from ref. [102], Elsevier 2022.

The aim of this study was to assess the toxicity of different surface-modified zinc oxide nanoparticles (ZnO NPs) in the developmental stages of zebrafish. Additionally, the study sought to investigate the toxicological mechanisms of these modified ZnO NPs within liver tissues [99]. Two types of ZnO NPs with amino (NH_2_-ZnO NPs) or carboxyl (COOH-ZnO NPs) modifications were tested in zebrafish embryos and larvae. Both types of ZnO NPs showed severe toxicity, with NH_2_-ZnO NPs demonstrating higher toxicity at lower concentrations [100]. Prolonged exposure to NH_2-_ZnO NPs resulted in a noteworthy reduction in zebrafish body length. Hazard rankings were computed for bulk ZnO and ZnO NPs, revealing hazard levels that followed a descending order of NH_2_-ZnO NPs, COOH-ZnO NPs, and bulk ZnO. Notably, NH_2_-ZnO NPs induced an accumulation of reactive oxygen species (ROS) in developing liver tissue, triggering the upregulation of autophagy-related genes and proteins. As a result of this cascade, there was a subsequent occurrence of liver cell apoptosis and a reduction in liver sizes. These findings provide insights into the potential risks associated with various surface modifications of zinc oxide nanoparticles (ZnO NPs) in aquatic environments. Additionally, they offer guidance for the selection of appropriate ZnO NPs for future industrial applications. Notably, NH_2_-coated NPs exhibited increased toxicity compared to the COOH group. The study also identified several phenotypic abnormalities induced by ZnO NPs, including tail abnormality (TL), hyperemia (HY), axial curvature (AC), pericardial edema (PE), yolk sac edema (YSE), and ocular malformations. Among these malformations, pericardial edema and yolk sac edema were identified as major abnormalities. The NH_2_-treated groups demonstrated particularly high levels of teratogenicity, as illustrated in Figure 11A [91].

Extended exposure to NH_2_-ZnO NPs led to a significant reduction in the body length of zebrafish. Hazard assessments were conducted for bulk ZnO and ZnO NPs, revealing a hazard ranking that decreased in the order of NH2-ZnO NPs, COOH-ZnO NPs, and bulk ZnO. Importantly, NH_2_-ZnO NPs induced the accumulation of reactive oxygen species (ROS) within developing liver tissue, which in turn triggered the upregulation of genes and proteins associated with autophagy. Consequently, this molecular cascade led to apoptosis of liver cells and a reduction in liver size. These findings provide valuable insights into the potential environmental risks associated with different surface modifications of ZnO NPs and offer guidance for the selection of appropriate ZnO NPs in future industrial applications.

In a recent investigation, the influence of simulated solar light (SSL) on the toxicity and dissolution characteristics of various zinc oxide nanoparticles (ZnO NPs) with diverse properties, including size, surface coatings, dopant chemistry, and aspect ratios, was examined using both a fish cell line and zebrafish embryos as model systems. The findings indicated an increase in toxicity and organism mortality when exposed to both SSL and ZnO NPs. This toxicity was attributed to the release of zinc ions and the generation of reactive oxygen species. However, surface modifications with substances such as poly(methacrylic acid) (PMAA), silica, or serum coatings were observed to alleviate the toxicity. Specifically, ZnO NPs with a serum coating had no significant impact on any of the cytotoxicity parameters, whether in dark or SSL conditions. These findings suggest that exposure to light can enhance the toxicity of ZnO NPs and that altering their surface chemistry can alleviate their toxicity, as illustrated in Figure 11B [101].

The understanding of interactions between microplastics (MPs) and ZnO NPs is limited [103,104], so a study was conducted to investigate the effects of exposure to MPs, ZnO NPs, and Zn^2+^ on zebrafish larvae [102]. This study examined the impact of exposing zebrafish larvae to various substances, including 500 μg/L of polystyrene MPs, 1200 μg/L of ZnO NPs, 500 μg/L of dissolved Zn^2+^ from ZnSO_4_, and combinations of MPs with ZnO NPs or ZnSO_4_. The results revealed that ZnO particles adhered to the surfaces of MPs, leading to increased transport of Zn into the larvae. Exposure to either MPs or ZnO NPs individually resulted in growth inhibition, oxidative stress, apoptosis, and disruption of the growth hormone (GH)/insulin-like growth factor (IGF) axis in F0 larvae. These effects were further exacerbated by co-exposure to both MPs and ZnO NPs. Co-exposure also increased the number of apoptotic cells in the gills and esophagus compared to exposure to MPs or ZnO NPs alone. In the F1 larvae from parents exposed to these substances, reduced growth, decreased antioxidant capacity, and downregulation of the GH/IGF axis were observed, but these effects were only evident in larvae exposed to a combination of MPs and ZnO NPs. Dissolved Zn^2+^ had a protective effect against MP toxicity, but it was insufficient to mitigate the adverse effects of ZnO particles. In summary, the findings suggest that interactions between particles, rather than the release of Zn^2+^ from ZnO NPs, amplified the toxicity of microplastics in early-stage zebrafish and their unexposed offspring, as depicted in Figure 11C [102]. 

### 3.3. Copper Oxide Nanoparticles

**Figure 12:**
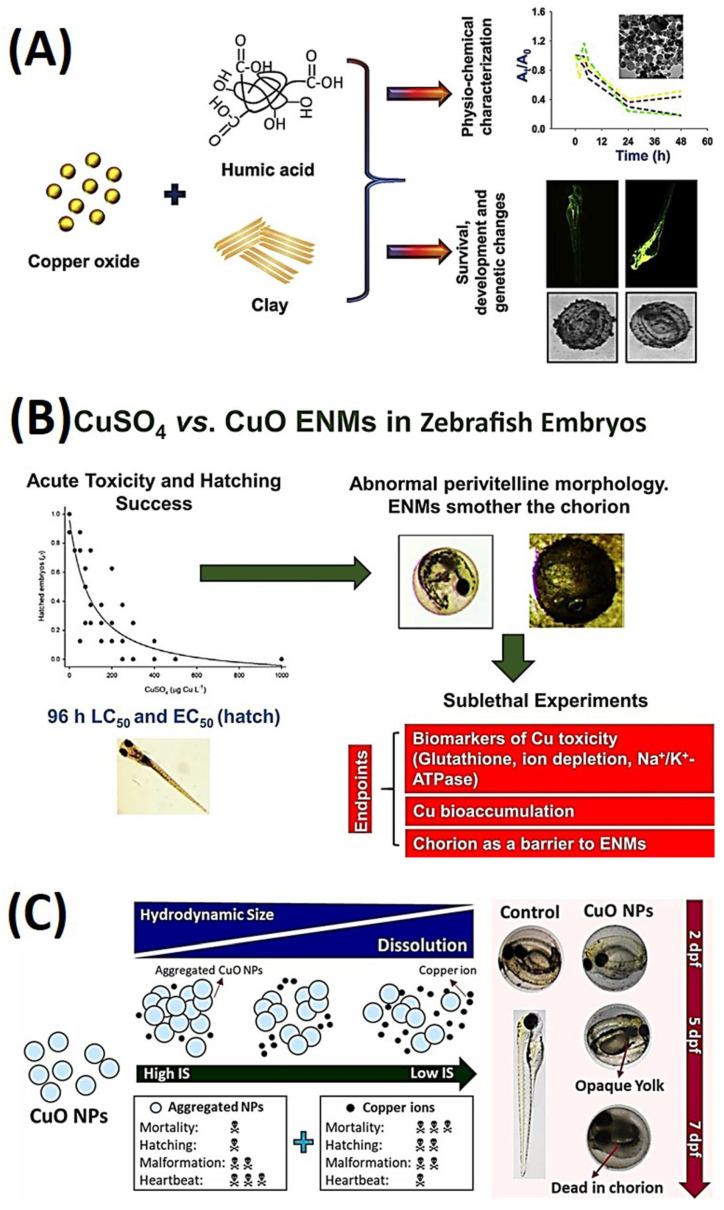
(**A**) Abiotic factors affect the stability and aggregation of copper oxide nanoparticles (CuO NPs), impacting zebrafish embryo development. Humic acid (HA) reduced bioavailability, while clay minerals caused heteroagglomeration. CuO NPs altered gene expressions related to zebrafish development, but a combination of HA and clay had a protective effect. Reproduced with permission from ref. [105], Elsevier 2019. (**B**) Comparative toxicity of copper sulfate (CuSO_4_) and CuO-engineered nanomaterials (ENMs) on early-life-stage zebrafish embryos revealed that CuO ENMs were orders of magnitude less toxic than CuSO_4_, with differences observed in their uptake, impacts on ion regulation, and antioxidant responses. Reproduced with permission from ref. [106], Elsevier 2023. (**C**) Effect of ionic strength on CuO NPs: impact on toxicity and bioaccumulation in zebrafish embryos. Reproduced with permission from ref. [107], Elsevier 2021.

The escalating utilization of copper oxide nanoparticles (CuO NPs) in commercial and industrial applications has prompted concerns regarding their potential release into aquatic environments. Consequently, researchers have conducted investigations to evaluate their impacts on both human and environmental systems. The objective of this study was to gain a comprehensive understanding of how abiotic factors affect the stability and aggregation of CuO NPs and their association with the development of zebrafish embryos. The research findings revealed that the presence of humic acid (HA) led to a reduction in the bioavailability of CuO NPs, while the introduction of clay minerals resulted in heteroagglomeration. Furthermore, the study observed significant changes in the expression of genes responsible for the development of the dorsoventral axis and neural network in zebrafish embryos when exposed to CuO NPs, CuO NPs + HA, and CuO NPs + clay. Particularly noteworthy was the protective effect on the development of zebrafish embryos when HA and clay were combined. These results offer new insights into the interactions between nanoparticles and abiotic factors, as well as their collective influence on genetic markers during the development of zebrafish embryos, as illustrated in Figure 12A [105].

The mechanisms underlying the toxicity of engineered nanomaterials (ENMs) to the early life stages of freshwater fish, as well as their relative hazard compared to dissolved metals, remain incompletely understood. To address this knowledge gap, zebrafish embryos were subjected to lethal concentrations of copper sulfate (CuSO_4_) or CuO ENMs with an approximate primary size of 15 nm. Subsequently, sublethal effects were investigated at a concentration corresponding to 10% of the lethal concentration (LC_10_) over a 96 h duration [106]. The study results demonstrated that the 96 h LC50 for CuSO_4_ was 303 ± 14 µg Cu/L, whereas for CuO ENMs, it was 53 ± 9.9 mg/L. This substantial difference indicated that ENMs were significantly less toxic than metal salt. Hatching success median effective concentrations (EC_50_) were found to be 76 ± 11 µg Cu/L for CuSO_4_ and 0.34 ± 0.78 mg/L for CuO ENMs. Failure to hatch in the CuSO_4_ group was associated with the presence of bubbles and foam-like perivitelline fluid, while in the CuO ENMs group, it was linked to particulate material covering the chorion. Regarding sublethal exposure, approximately 42% of the total copper from CuSO_4_ was internalized, whereas, for ENMs, nearly all (94%) of the total copper remained associated with the chorion. This observation suggested that the chorion provided effective short-term protection for embryos against ENMs. Both forms of copper exposure led to the depletion of sodium (Na^+^) and calcium (Ca^2+^) within the embryos, with CuSO_4_ also causing some inhibition of sodium pump (Na^+^/K^+^-ATPase) activity. Furthermore, both types of copper exposure resulted in a decrease in total glutathione (tGSH) within the embryos, although they did not induce superoxide dismutase (SOD) activity. In summary, CuSO_4_ was found to be more toxic than CuO ENMs to early-life-stage zebrafish, and there were subtle differences in the exposure and toxicological mechanisms between the two substances, as depicted in Figure 12B [106].

In another study, the emphasis was on investigating the transformation of copper oxide nanoparticles (CuO NPs) in aquatic environments with different levels of ionic strength (IS). The study aimed to understand how these alterations influenced copper toxicity and bioaccumulation in zebrafish embryos [108]. To replicate different ionic strength (IS) conditions resembling surface water, groundwater, and wastewater (representing low-, mid-, and high-IS water, respectively), a series of experiments were conducted. The results indicated that as IS decreased from 54 to 1.5 mM, zebrafish larval mortality increased from 21.3% to 33.3% at the highest CuO concentration of 10 mg/L. In low-IS water, the study also observed the highest occurrence of delayed hatching embryos (81.3%) and yolk deformations (36.3%). Furthermore, copper bioaccumulation was significantly higher in larvae exposed to low-IS water (15%) compared to those exposed to high-IS water (35%). The research also revealed that CuO NPs in low-IS water rapidly formed relatively small aggregates with high copper dissolution, posing a substantial risk to aquatic organisms. Conversely, aggregated CuO NPs (>500 nm) in mid- and high-IS waters were effectively prevented from reaching the embryos and were predominantly found in the body axis and tail. These findings emphasize the potential hazards associated with CuO NPs in low-IS solutions, where they quickly form aggregates and result in elevated copper uptake, especially in the hearts of the larvae, as depicted in Figure 12C [107].

### 3.4. Iron Oxide Nanoparticles

**Figure 13:**
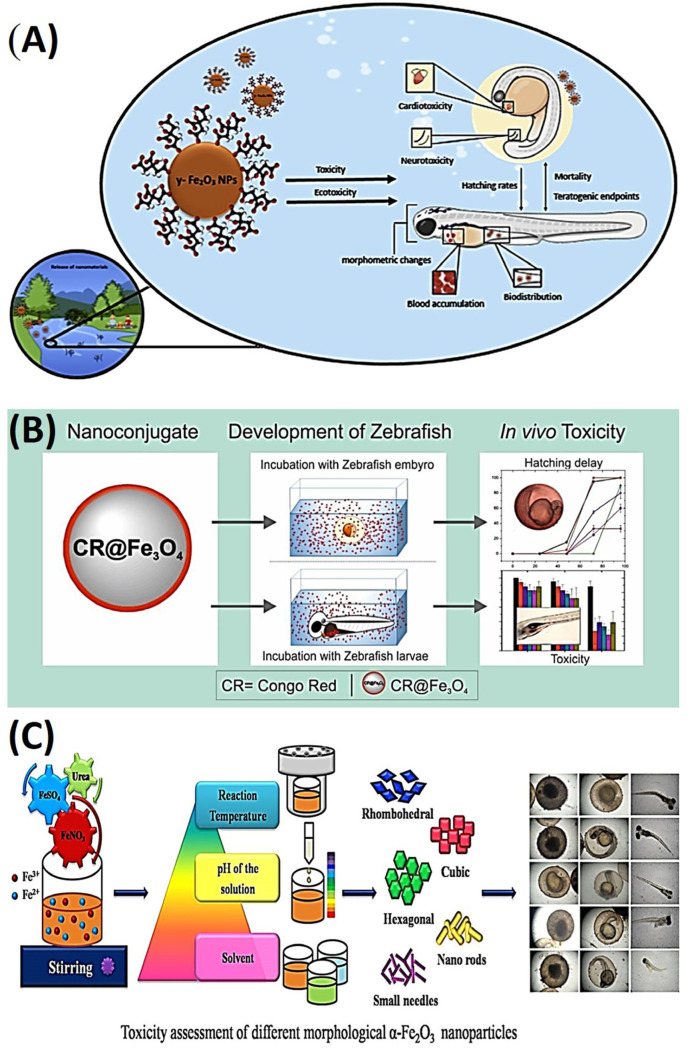
(**A**) Developmental toxicity of iron oxide nanoparticles (IONPs) and dissolved iron in zebrafish embryos. IONPs accumulated on the chorion and caused sublethal cardiotoxic effects. Semi-static exposure induced higher embryotoxicity than static exposure. Reproduced with permission from ref. [110], Elsevier 2020. (**B**) Evaluating the developmental toxicity of CR@Fe_3_O_4_ NPs toward zebrafish. Reproduced with permission from ref. [111], Elsevier 2020. (**C**) Schematic illustration of toxicological assessment of different IONPs using a zebrafish model. Reproduced with permission from ref. [112], Elsevier 2019.

In a recent study, the objective was to investigate the developmental toxicities of citrate-functionalized iron oxide nanoparticles (IONPs) and their dissolved counterparts in zebrafish [109]. The research aimed to comprehend the impact of IONPs, extensively utilized in medical and environmental applications, on the early developmental stages of fish. Throughout the study, fish embryos were subjected to different concentrations of two distinct forms of iron, spanning from 0.3 to 10 mg/L, over a 144 h duration. The exposure was carried out under both static and semi-static conditions, and a negative control group was included for comparison. Despite exhibiting lower levels of embryotoxicity compared to iron ions in both exposure conditions, citrate-functionalized iron oxide nanoparticles (IONPs) did induce sublethal effects, primarily in the form of cardiotoxic issues such as reduced heart rate, blood accumulation in the heart, and pericardial edema. Notably, it was observed that semi-static exposure to both forms of iron resulted in higher levels of embryotoxicity compared to static exposure. This suggests that the nanotoxicity affecting the early developmental stages of fish is influenced by the chosen exposure system. This study represents a pioneering effort to investigate the influence of exposure conditions on the developmental toxicity of IONPs in fish species, as illustrated in Figure 13A [110].

In a recent study, the concentration-dependent toxicity of iron oxide (IO) nanoparticles, specifically magnetite (Fe_3_O_4_) NPs, in zebrafish was evaluated. These nanoparticles are frequently used as carriers for diagnostic and therapeutic agents in biomedical applications. To monitor their uptake and accumulation in zebrafish during early developmental stages, the particles were functionalized with a fluorescent dye known as Congo red (CR). In this study, zebrafish embryos and larvae were exposed to varying concentrations of CR@Fe_3_O_4_ conjugates (ranging from 100 to 800 μg/mL) for periods between 4 to 96 h post fertilization (hpf). The research examined parameters such as mortality, hatching rate, and the extent of whole-embryo cellular death. The findings indicated that lower concentrations of these nanoparticles did not result in adverse developmental toxicity during the embryonic and larval stages of zebrafish. However, exposure to a high concentration (800 μg/mL) led to increased mortality and delays in the hatching process. Furthermore, the study revealed that CR@Fe_3_O_4_ had a more pronounced toxic effect on zebrafish larvae, suggesting a greater bioavailability of the nanoparticles during this developmental stage. Another investigation utilized CR@Fe_3_O_4_ NPs as an optical probe to explore the potential nanotoxicological effects of Fe_3_O_4_ NPs in vivo systems, contributing to our understanding of nanotoxicity, as depicted in Figure 13B [111]. In another study, hematite (α-Fe_2_O_3_) NPs were synthesized via a hydro-solvothermal technique under various conditions, [113] resulting in different sizes and shapes. The synthesized NPs were characterized using X-ray diffraction (XRD), field emission scanning electron microscopy (FESEM) with energy-dispersive X-ray (EDX), FTIR, and vibrating sample magnetometry, which revealed that the size and shape could be modified by adjusting synthetic parameters such as the reaction temperature, pH, and solvents used. In the pursuit of ecological toxicity assessment, zebrafish embryos underwent exposure to varying concentrations of α-Fe_2_O_3_ NPs, and their physical attributes were closely observed. The evaluation encompassed hatching rates, survival percentages, heartbeats, and body length measurements. Lower concentrations yielded minor alterations, while higher concentrations resulted in noticeable malformations, in contrast to the control group. These findings underline the significance of both nanoparticle size and concentration in determining the toxicity of α-Fe_2_O_3_ NPs towards zebrafish, as illustrated in Figure 13C [112].

### 3.5. Graphene Oxide

**Figure 14:**
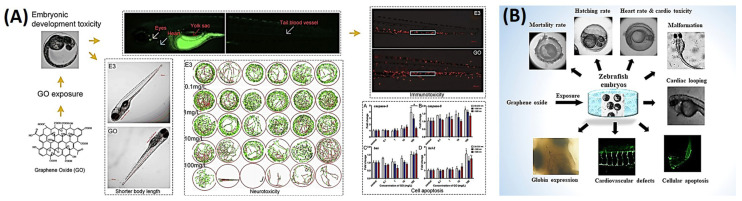
(**A**) Graphene oxide (GO) particles of different sizes cause developmental toxicity in zebrafish, leading to physical and behavioral alterations, altered gene expressions, and oxidative stress. Reproduced with permission from ref. [114], Elsevier 2020. (**B**) Assessing the effects of GO on cardiovascular defects and embryonic development in zebrafish embryos, it was found that GO at high concentrations could cause significant embryonic mortality, cardiotoxicity, and cardiovascular defects. Reproduced with permission from ref. [115], Elsevier 2019.

The utilization of graphene oxide (GO) is increasingly widespread in various fields, but its release into the environment poses potential ecological and environmental risks. This study aimed to explore the effects of different-sized GO particles (50~200, <500, and >500 nm) on zebrafish during early developmental stages (4~124 hpf). The results revealed that GO particles accumulated in various organs of fish larvae, including the eyes, heart, yolk sac, and blood vessels, leading to several toxic effects. These effects included delayed hatching, reduced body length, altered heart rate and blood flow, changes in swimming activity and responses to photoperiod stimulation, and increased activities of enzymes and genes related to oxidative stress and apoptosis. The toxicity effects were found to be correlated with the concentration of GO and were also associated with the size of the GO particles. These findings indicate that waterborne exposure to GO can pose significant ecological and health risks, emphasizing the importance of considering such risks when using GO materials, as illustrated in Figure 14A [114].

GO boasts a wide array of engineering applications spanning electronics, energy storage, pharmaceuticals, nanomedicine, environmental remediation, and biotechnology, all thanks to its unique physicochemical properties. However, concerns linger regarding its potential ecological and health risks. While the developmental toxicity of GO in zebrafish has been extensively studied, its impact on living organisms with specific cardiovascular defects remains unclear. Hence, this study sought to assess the toxicity of GO during embryonic development and its effect on cardiovascular defects in zebrafish embryos, serving as an in vivo animal model [116]. The findings revealed that at low concentrations (0.1–0.3 mg/mL), graphene oxide (GO) did not hinder embryonic development. However, higher concentrations (0.4–1 mg/mL) resulted in significant outcomes, including increased embryonic mortality, delayed hatching, cardiotoxicity, the emergence of cardiovascular defects, retardation of cardiac looping, elevated apoptosis, and reduced hemoglobinization. These results provide valuable insights into the ecotoxicological implications of GO and its biosafety concerning environmental concentrations. Furthermore, they contribute to our understanding of the environmental risks associated with GO, addressing both ecological and human health concerns, as depicted in Figure 14B [115].

### 3.6. Copper Sulfide Nanoparticles

Metal and metal oxide nanoparticles (NPs) are commonly detoxified through sulfidation. Copper sulfide (CuS) NPs are generally considered relatively harmless to aquatic wildlife due to their low solubility in water. However, the use of hypochlorite, a common disinfectant in wastewater treatment, may increase the potential risks of toxicity to aquatic life and human health by promoting the dissolution of CuS NPs in water. A recent study has shed light on the impact of hypochlorite disinfection on the chemical stability of CuS NPs and the exacerbated toxicity of CuS NPs resulting from the dissolution induced by hypochlorite [117]. The results revealed that hypochlorite oxidation compromised the chemical stability of CuS NPs and heightened the bioavailability of dissolved copper in zebrafish embryos. While zebrafish embryos did not display significant adverse effects when exposed to CuS NPs alone, the combination of CuS NPs and hypochlorite led to substantial developmental toxicity in early-stage zebrafish. These findings were further substantiated through metabolomics and in vivo experiments, demonstrating that the intensified toxicity was attributed to the induction of oxidative stress and membrane damage. This study underscores the importance of considering the transformations of nanoparticles in water treatment processes when delving into nanotoxicology. It also suggests that CuS NPs may pose a heightened risk to aquatic organisms when subjected to hypochlorite disinfection.

Another study focused on the extracellular synthesis and characterization of CuS NPs using *Aeromonas hydrophila*, as well as their potential biological applications as antibacterial, anti-inflammatory, and antioxidant agents. The toxicity of CuS NPs was also evaluated using zebrafish as an animal model. In the synthesis process, precursor copper sulfates were added to the culture supernatant of *A. hydrophila* [118]. Synthesized NPs were characterized using UV-visible spectrophotometry, which revealed a peak at 307 nm due to the reduction process. FTIR was used to identify functional groups such as carboxylic acid, alcohols, alkanes, and nitro compounds associated with CuS NPs. AFM was used to study the topography of CuS, and the SEM revealed that the NPs were about 200 nm in size and had agglomerated structures. Overall, the CuS NPs characterized in this study show potential as therapeutic agents with antibacterial, antioxidant, and anti-inflammatory properties [119].

The toxicity of CuS NPs remains poorly understood despite significant research on copper-based NPs. Given the increasing use of CuS-based nanomaterials in biomedical engineering, it is imperative to investigate their potential toxicity and biological effects. In this study, zebrafish embryos were exposed to varying concentrations of polymer-modified CuS nanoclusters (PATA3-C4@CuS) to assess their impact on embryo development [120]. The results revealed that PATA3-C4@CuS concentrations exceeding 1 mg/L resulted in abnormal phenotypes, increased mortality, reduced hatching rates, and inhibited swim bladder inflation. Exposure to PATA3-C4@CuS also led to alterations in the expression patterns of genes associated with cell migration and cardiac development and function. Furthermore, PATA3-C4@CuS caused damage to the ventral projection of primary motor neurons, ultimately diminishing locomotion ability. Consequently, this study suggests that functional PATA3-C4@CuS may disrupt cell migration during gastrulation, influence cardiac development and function, and reduce locomotor activity. Despite extensive research on CuO NPs, which has provided a wealth of toxicity information, our understanding of the toxicity of CuS NPs remains limited [121].

### 3.7. Zirconium Dioxide Nanoparticles

**Figure 15:**
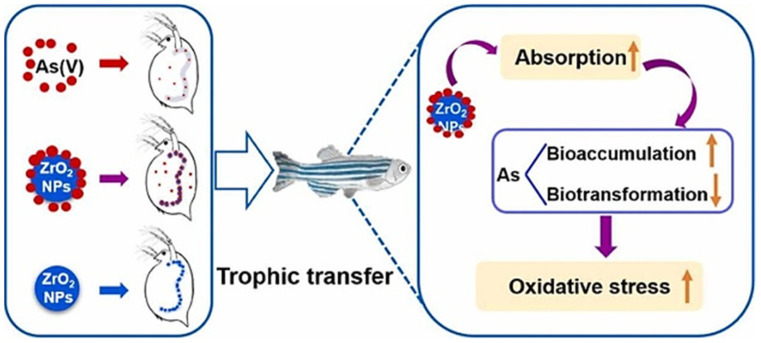
Zirconium dioxide nanoparticles (ZrO_2_ NPs) enhance arsenate (As(V)) accumulation, transformation, and toxicity in *Daphnia magna* and zebrafish. Co-exposure to As(V) and ZrO_2_ NPs induced gene expression changes and amplified ecological risks in the aquatic food chain. Reproduced with permission from ref. [123], Elsevier 2022.

Recent work delved into the influence of zirconium dioxide (ZrO_2_) NPs on the accumulation, trophic transfer, transformation, and detoxification processes of arsenate (As(V)) within the aquatic food chain, with a specific focus on *Daphnia magna* (water flea) and *Danio rerio* (zebrafish) [122]. The findings revealed that ZrO_2_ NPs heightened the absorption of both total arsenic and As(V) in the tissues of both water fleas and zebrafish. Additionally, these nanoparticles facilitated the conversion of inorganic arsenic (iAs) into monomethylated acid (MMA) while diminishing the production of arsenobetaine (AsB), consequently elevating iAs levels. Moreover, co-exposure to As(V) and ZrO_2_ NPs upregulated genes associated with absorption (e.g., *aqp7*), biotransformation (e.g., *gst* and *gss*), and detoxification as well as oxidative stress (e.g., *mt2*, *cat*, *sod1*, and *sod2*). These genetic responses led to heightened detrimental effects on both water fleas and zebrafish, exacerbating the ecological risks associated with As(V) within the aquatic food chain. This study underscores the imperative to consider the cumulative toxicity of As(V) and ZrO_2_ NPs in aquatic environments, as depicted in Figure 15 [123].

Zirconium dioxide nanoparticles (ZrO_2_ NPs) find a wide range of beneficial applications in biomedical and industrial fields. To comprehend their effects on embryonic development in zebrafish, ZrO_2_ NPs synthesized by the solvent-gelation method were administered to zebrafish embryos at different concentrations during 24 to 96 h post-fertilization (hpf) to observe developmental toxicity. The findings revealed that exposure to concentrations ranging from 0.5 to 1 μg/mL of ZrO_2_ NPs resulted in mortality, hatching delay, and malformations, including a bent axis, bent tail, spinal cord curvature, and yolk-sac and pericardial edema. Embryos exposed to 1 μg/mL of ZrO_2_ NPs showed a typical phenotype of unhatched dead embryos. This study highlighted the potential biomedical toxicological effects of ZrO_2_ NPs and supported the safety evaluation and synthesis of these NPs. It is one of the first reports on the developmental toxicity of ZrO_2_ NPs in aquatic environments, and the results suggested that even lower concentrations of these NPs can be highly toxic to embryonic zebrafish [124].

### 3.8. Molybdenum Disulfide Nanosheets

**Figure 16:**
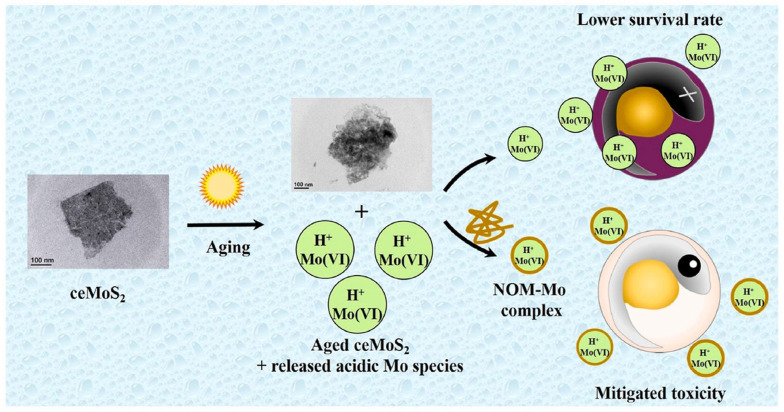
Aging of chemically exfoliated molybdenum disulfide (ceMoS_2_) nanosheets led to the release of ionic aging products, including acidic Mo species, and reduced survival rates in embryonic zebrafish, which could be mitigated by complexation with natural organic matter (NOM). Reproduced with permission from ref. [126], Elsevier 2022.

One study aimed to investigate the effects of environmental processes on the biological impacts of chemically exfoliated (ce) molybdenum disulfide (MoS_2_) nanosheets (NSs) on aquatic organisms. The research found that the bioavailability and chemical speciation transitions occurring during aging were critical factors that influenced the toxicity of ceMoS_2_ NSs [125]. Aged ceMoS_2_, which had been exposed to sunlight and dark ambient conditions, released ionic aging products, including acidic Mo species, leading to lower survival rates in embryonic zebrafish compared to freshly prepared ceMoS_2_. The study unveiled that soluble molybdenum, once released, engaged in interactions with natural organic matter (NOM), thus mitigating the toxicity induced by ceMoS_2_ to varying extents. Specifically, Suwannee River NOM demonstrated a significant reduction in the toxicity of aged ceMoS_2_, regardless of whether the conditions were dark or irradiated. This reduction was attributed to the formation of complexes between NOM and ionic Mo species, a conclusion substantiated by Mo K-edge X-ray absorption spectroscopy. These insights shed valuable light on the potential impacts of ceMoS_2_ on aquatic organisms and offer guidance for the safe utilization of MoS_2_ nanosheets, as depicted in Figure 16 [126]. In a separate investigation, the ecotoxicity of untreated mining effluent sourced from the largest molybdenum open-pit mine in China’s Qinling Mountains, along with its treated counterpart, was evaluated using zebrafish as the model organism. The study unearthed that the mining effluent qualified as acid mine drainage (AMD) and exhibited exceptionally high toxicity towards zebrafish, with a 96 h LC50 value of 3.80% (volume percentage) for the untreated effluent. Additionally, exposure to sublethal concentrations of the untreated effluent (1/50, 1/10, and 1/2 of the 96 h LC50) led to oxidative stress and disturbances in osmoregulation [127]. These effects were discerned through changes in enzymatic activities like superoxide dismutase and catalase, alterations in malondialdehyde levels, and the inhibition of Na^+^/K^+^-ATPase activity in the gills and muscles of zebrafish following 28 days of subchronic exposure. Furthermore, employing a neutralizer (NaOH) and activated carbon as an adsorbent in the treatment process ameliorated the acute lethality of the raw effluent. Based on these findings, the study recommended cost-effective endpoints—encompassing acute lethality and biochemical markers associated with oxidative stress and osmoregulatory disruption in zebrafish—as effective tools for assessing the toxicity of AMD such as the molybdenum mining effluent under examination. As a result of these findings, the study proposed strategies for the management of mining effluents. These included constraints on the discharge of both untreated and diluted effluents into nearby water bodies and the implementation of a comprehensive biomonitoring system to cover all mining drainage systems in the region [128]. A summary of nanomaterials and their models and toxicology assessments is shown in Table 1.

## 4. Challenges and Perspectives

Zebrafish have emerged as a valuable in vivo model for assessing the toxicity of nanomaterials, particularly in evaluating malformations and functional defects. However, the existing literature indicates a notable gap in nanomaterial-based immunotoxicity assays. Additionally, conducting systematic embryo-based nanotoxicity assessments faces challenges due to the rapid developmental stages of zebrafish. Nonetheless, leveraging automation and advanced technologies can enhance nanotoxicity screening using zebrafish embryos. Nanomaterials find application in therapeutics, including drug delivery and antimicrobial treatments, emphasizing the importance of understanding their absorption, distribution, metabolism, and excretion (ADME) properties. Uncertainties persist regarding how ADME assays will perform in zebrafish models post-nano-drug delivery. The zebrafish stands as a robust in vivo model for exploring nanomaterial toxicity, bolstered by an array of molecular biology techniques and transgenic lines. The availability of zebrafish microarrays and extensive genomic resources further enhances its versatility for toxicogenomic investigations of nanomaterials. Analyzing gene and protein expressions during zebrafish development holds promise for elucidating the complex issue of nanomaterial toxicity. While high-throughput screening systems involving larval zebrafish are already in use for nanomaterial toxicity studies, there remains substantial untapped potential for advancing such assays with this model organism.

## Figures and Tables

**Table 1:** Summary of nanomaterials, models, and toxicology assessments.

Nanomaterial	Models	Toxicology	Reference
Au	Zebrafish	Size directly proportional to toxicity	[30]
ZnO, CuO, and NiO	Zebrafish	Delayed hatching of embryos	[36]
TMs doped Co_3_O_4_ and PdO-Co_3_O_4_	Zebrafish	Severe skin damage	[37]
Ag	Zebrafish	Size dependent intestinal toxicity	[57]
Ag	Zebrafish	Size dependent toxicity	[58]
Ag	Zebrafish	Dosage dependent toxicity	[59]
RU-Ag	Zebrafish	Malformation, imbalanced heart rate, and severe morphological changes	[60]
Cu	Zebrafish	Decreased pigmentation at 85 ppm	[69]
Cu	Zebrafish	Intestinal damage by ROS	[67]
CuO	Zebrafish	DNA damage and cytotoxicity	[71]
Pt	*Pseudokirchneriella subcapitata* *Chlamydomonas reinhardtii*	Growth rate suppression and oxidative stress	[76]
Au-Ag	Early-stage zebrafish embryos	Superior biocompatibility and decreased cytotoxicity	[78]
Ru-Fc	Zebrafish	Mitochondrial malfunction, ER stress, and necroptotic cell death	[79]
Fe-ZnO	Zebrafish and Mouse	Reduced inflammation and lactate dehydrogenase release	[80]
NH_2_-ZnO	Zebrafish	Induced hepatotoxicity by ROS	[91]
TiO_2_	Zebrafish	1.0 mg/L nano-TiO_2_ significantly reduced the body length and weight	[95]
TiO_2_	Zebrafish	Caused neurotoxicity and abnormal growth	[96]
TBPH-TiO_2_	Zebrafish	Reduced toxicity and anomalies	[97]
ZnO-SCs	Zebrafish	Reduced toxicity and anomalies	[101]
MPs and ZnO	Zebrafish	Growth inhibition, oxidative stress, apoptosis, and disruption of the growth hormone/insulin growth factor axis	[102]
CuO-clay-HA	Zebrafish	Reduced toxicity and anomalies	[105]
CuSO_4_-CuO ENMs	Zebrafish	Reduced toxicity and anomalies by limiting oxidative stress	[106]
CuO	Zebrafish	The highest number of delayed hatching embryos (81.3%) and opaque yolk deformation (36.3%)	[107]
IO	Zebrafish	Reduced heartbeat, blood accumulation in the heart, and pericardial edema	[110]
Congo red@IO	Zebrafish	Delayed hatching and increased mortality	[111]
α-IO	Zebrafish	Concentration dependent toxicity	[112]
GO	Zebrafish	Size dependent developmental toxicity	[114]
GO	Zebrafish	Delayed hatching, reduced body length, altered heart rate and blood flow, changes in swimming activity and responses to photoperiod stimulation, and increased activity of enzymes and genes related to oxidative stress and apoptosis.	[115]
ZrO_2_	*Daphna magna* and Zebrafish	DNA damage and toxic anomalies	[123]
MoS_2_	Zebrafish	Reduced toxicity with NOM combination	[126]

Abbreviations: Au, gold; Ag, silver; Cu, copper; Pt, platinum; As, arsenic; Co/Ni, cobalt/nickel; Ru-Fc, ruthenium–ferrocene; TiO_2_, titanium dioxide; MPs and ZnO, microplastics and zinc oxide; CuO, copper oxide; NiO, nickel oxide; TMs, transition metals; Co_3_O_4_, cobalt oxide; PdO, palladium oxide; IO, iron oxide; GO, graphene oxide; CuS, copper sulfate; ZrO_2_, zirconium dioxide; MoS_2_, molybdenum disulfide; NPs, nanoparticles; ROS, reactive oxygen species; ER, endoplasmic reticulum; UPR, unfolded protein response; NOM, natural organic matter; HA, humic acid; ENMs, engineered nanomaterials; TBPH, bis(2-ethylhexyl)-2,3,4,5-tetrabromophthalate; CuSO_4_, copper sulfate; SCs, surface coatings; RU, *Rumex acetosa*;.

## Data Availability

No new data were created or analyzed in this study. Data sharing is not applicable to this article.
